# Lipids Extracted from *Aptocyclus ventricosus* Eggs Possess Immunoregulatory Effects on RAW264.7 Cells by Activating the MAPK and NF-κB Signaling Pathways

**DOI:** 10.3390/md22080368

**Published:** 2024-08-13

**Authors:** Seul Gi Lee, Weerawan Rod-in, Jun Jae Jung, Seok Kyu Jung, Sang-min Lee, Woo Jung Park

**Affiliations:** 1 Department of Wellness-Bio Industry, Gangneung-Wonju National University, Gangneung 25457, Gangwon, Republic of Korea; tkrhk093@gmail.com; 2 Department of Marine Bio Food Science, Gangneung-Wonju National University, Gangneung 25457, Gangwon, Republic of Korea; weerawanro@nu.ac.th; 3 Department of Agricultural Science, Faculty of Agriculture Natural Resources and Environment, Naresuan University, Phitsanulok 65000, Thailand; 4 Center of Excellence in Research for Agricultural Biotechnology, Naresuan University, Phitsanulok 65000, Thailand; 5 East Coast Life Sciences Institute, Gangneung-Wonju National University, Gangneung 25457, Gangwon, Republic of Korea; gpgp444@gmail.com; 6 Department of Horticultural Science, Kongju National University, Yesan-gun 32439, Chungcheonnam-do, Republic of Korea; jungsk@kongju.ac.kr; 7 Department of Aquatic Life Medicine, Gangneung-Wonju National University, Gangneung 25457, Gangwon, Republic of Korea; smlee@gwnu.ac.kr; 8 KBIoRANCh Co., Ltd., Gangwon-do, Gangneung 25457, Republic of Korea

**Keywords:** lipids, cytokines, macrophages, inflammation, *A. ventricosus*

## Abstract

This study was conducted to evaluate the potential anti-inflammatory and immune-enhancement properties of lipids derived from *Aptocyclus ventricosus* eggs on RAW264.7 cells. Firstly, we determined the fatty acid compositions of *A. ventricosus* lipids by performing gas chromatography analysis. The results showed that *A. ventricosus* lipids contained saturated fatty acids (24.37%), monounsaturated fatty acids (20.90%), and polyunsaturated fatty acids (54.73%). They also contained notably high levels of DHA (25.91%) and EPA (22.05%) among the total fatty acids. Our results for the immune-associated biomarkers showed that *A. ventricosus* lipids had immune-enhancing effects on RAW264.7 cells. At the maximum dose of 300 µg/mL, *A. ventricosus* lipids generated NO (119.53%) and showed greater phagocytosis (63.69%) ability as compared with untreated cells. *A. ventricosus* lipids also upregulated the expression of *iNOS*, *IL-1β*, *IL-6*, and *TNF-α* genes and effectively upregulated the phosphorylation of MAPK (JNK, p38, and ERK) and NF-κB p65, indicating that these lipids could activate the MAPK and NF-κB pathways to stimulate macrophages in the immune system. Besides their immune-enhancing abilities, *A. ventricosus* lipids significantly inhibited LPS-induced RAW264.7 inflammatory responses via the NF-κB and MAPK pathways. The results indicated that these lipids significantly reduced LPS-induced NO production, showing a decrease from 86.95% to 38.89%. Additionally, these lipids downregulated the expression of genes associated with the immune response and strongly suppressed the CD86 molecule on the cell surface, which reduced from 39.25% to 33.80%. Collectively, these findings imply that lipids extracted from *A. ventricosus* eggs might have biological immunoregulatory effects. Thus, they might be considered promising immunomodulatory drugs and functional foods.

## 1. Introduction

Inflammation is one of the important immune regulation systems and a highly complex one that protects against external harm or tissue damage caused by various stimuli, including pathogens, toxic chemicals, mechanical substances, and autoimmune responses [1,2]. Numerous marine-derived compounds have immune-modulatory properties that can reduce inflammation while also stimulating immunity [3,4]. RAW264.7 is a macrophage cell line established from a tumor in a mouse induced with the Abelson murine leukemia virus. It is a useful model for examining immune activities of macrophages [5,6,7,8]. Activated macrophages are known to release multiple immunomodulatory factors such as nitric oxide (NO), inducible nitric oxide synthase, prostaglandin E_2_ (PGE_2_), cyclooxygenase (COX)-2, reactive oxygen species (ROS), interleukin-1β (IL-1β), IL-6, IL-10, IL-12, tumor necrosis factor-α (TNF-α), and monocyte chemoattractant protein-1 (MCP-1), which can serve as indicators of immunity [6,9,10,11]. Additionally, they can promote immunoregulatory effects by triggering the NF-κB and MAPK signaling pathways [6,11,12]. Moreover, many kinds of natural compounds have been revealed to affect the immunological responses of macrophages [6,9,11].

Fish-derived lipids are known to contain substantial quantities of omega-3 polyunsaturated fatty acids (PUFAs), particularly C22:6n-3 (docosahexaenoic acid, DHA) and C20:5n-3 (eicosapentaenoic acid, EPA) [13,14,15]. They can act as lipid mediator precursors and affect inflammatory and immunological responses [16]. EPA and DHA also show potent anti-inflammatory effects in THP-1-derived macrophages [17]. In studies using RAW264.7 cells, EPA has been shown to possess biological functions of immune modulation, such as immune enhancement and anti-inflammation, in diverse marine species [5,12]. Lipids and fatty acids (FAs) have a variety of naturally occurring effects. For example, they can prevent thrombosis, diabetes, obesity, cancer, inflammation, Alzheimer’s disease, and cardiovascular diseases [18,19]. Lipids extracted from *Ammodytes personatus* eggs contain high levels of PUFAs, which boost immunity and reduce inflammation by regulating inflammatory mediators, cytokines, surface expression, and intracellular signaling pathways [20]. Previous research has demonstrated that CD14, CD40, and CD86 with immunopotentiation activity on the surfaces of macrophages can be activated by fish lipids and FAs [8,9,20,21].

*Aptocyclus ventricosus* (smooth lumpsucker) is a species of cold-water fish belonging to the Cyclopteridae family. These fish live in the North Pacific, originating from the coast of the Korean Peninsula including water areas of the East Sea of Korea, the Sea of Okhotsk, and the Bering Sea [22,23]. It is an iteroparous gonochoristic species with an extremely high fertilization rate. Its fully ripe internal egg masses have significant economic value [24]. *A. ventricosus* has been a popular food in the Gangwon-do region in Korea for a long time, with people especially favoring the roe for its good texture [25]. The muscles and roe (eggs) of *A. ventricosus* contains the following fatty acids: C16:0, C18:0, C18:1n-9, C18:1n-7, C20:4n-6, EPA, and DHA. Its eggs contain higher amounts of EPA and DHA compared to those of other fish species [25]. Consuming fish with substantial n-3 PUFAs has been shown to prevent mortality from fetal development issues, cardiovascular disease, Alzheimer’s disease, and chronic inflammatory diseases [26,27]. However, most studies on *A. ventricosus* have focused on its development, distribution, genetic features, morphological characteristics, taxonomy, physiological features, and responses to environmental changes [22,24,28,29]. Information about the functional materials of *A. ventricosus* for human health is limited. Moreover, no studies related to *A. ventricosus* eggs as promising immunomodulatory drugs or functional foods have yet to be described. In particular, the biological activities of *A. ventricosus*, especially the immune-enhancing and anti-inflammatory effects of lipids from *A. ventricosus* eggs on macrophages, have not yet been reported. Hence, the current study aimed to assess the immunological regulatory effects of lipids derived from *A. ventricosus* eggs on RAW264.7 cells.

## 2. Results

### 2.1. Analysis of Fatty Acids (FAs) in A. ventricosus Lipids

**Figure 1:**
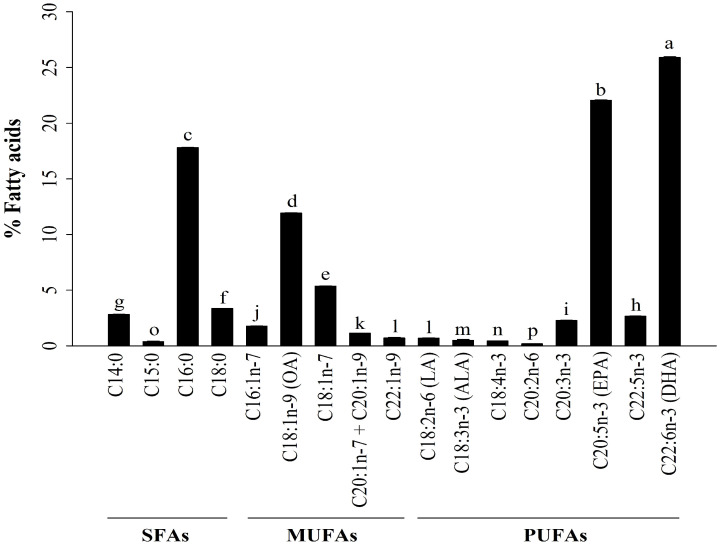
Compositions of FAs in *A. ventricosus* lipids (% of all detected FAs). Values are shown as the mean ± standard deviation (SD) (*n* = 5). Significant changes in fatty acid levels are shown by different superscript lowercase letters (*p* < 0.05).

In the present study, the GC-FID method was applied to determine the FA profiles of lipids derived from *A. ventricosus* eggs. Figure 1 depicts the FA compositions of *A. ventricosus* lipids which include saturated fatty acids (SFAs), monounsaturated fatty acids (MUFAs), and PUFAs. The FA compositions of *A. ventricosus* lipids included SFAs (24.37 ± 0.06%), MUFAs (20.90 ± 0.06%), and PUFAs (54.73 ± 0.90%). Among the SFAs present in *A. ventricosus* lipids, myristic acid (C14:0), palmitic acid (C16:0), and stearic acid (C18:0) accounted for 2.82%, 17.81%, and 3.37%, respectively. Among the MUFAs, oleic acid (C18:1n-9) and C18:1n-7 accounted for 11.94% and 5.35%, respectively. Among the PUFAs, DHA (C22:6n-3), EPA (C20:5n-3), docosapentaenoic acid (C22:5n-3), and eicosatrienoic acid (C20:3n-3 cis-11) accounted for 25.91%, 22.05%, 2.66%, and 2.28%, respectively.

### 2.2. Cytotoxic Effect of A. ventricosus Lipids on Macrophages

**Figure 2:**
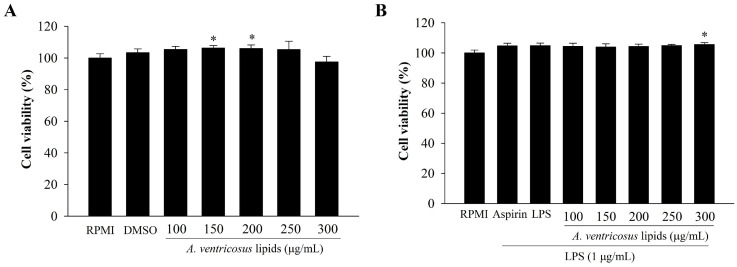
Effects of *A. ventricosus* lipids on cell viability of RAW264.7 cells without LPS stimulation (**A**) or with LPS stimulation (**B**). Values are shown as the mean ± SD (*n* = 3). * *p* < 0.05, indicating statistical differences compared to cells treated with RPMI.

The cytotoxic influence of *A. ventricosus* lipids on RAW264.7 macrophages was determined using an EZ-Cytox Cell Viability Assay Kit. The results are displayed in Figure 2. No treatment was cytotoxic to RAW264.7 cells, as assessed using untreated cells (RPMI) as the control. Figure 2A shows the cell viability upon treatment with *A. ventricosus* lipids without LPS stimulation. The results show that cell viability after treatment with DMSO (1%) or *A. ventricosus* lipids at 100, 250, or 300 µg/mL was not drastically different from that of RPMI. Although cell viability after treatment with A. *ventricosus* lipids at 150 or 200 µg/mL significantly improved cell viability compared to RPMI, no cytotoxicity was observed. For LPS-induced RAW264.7 cells, cell viability was not significantly different among the treatments except for the group treated with *A. ventricosus* lipids at the highest concentration (300 µg/mL) (Figure 2B). Likewise, LPS (1 µg/mL), aspirin (200 µg/mL), and the control showed no cytotoxicity. Therefore, 100–300 µg/mL of *A. ventricosus* lipids were determined to be the optimal concentrations for subsequent experiments.

### 2.3. Effects of A. ventricosus Lipids on Phagocytosis of Macrophages

**Figure 3:**
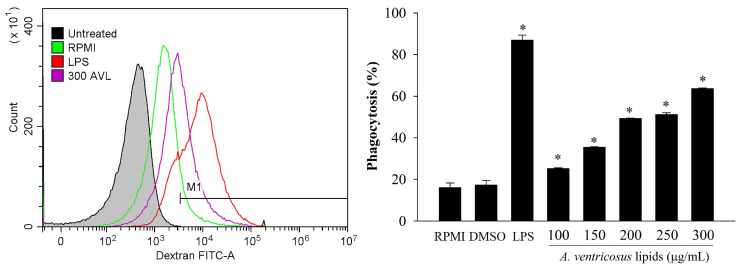
Effects of *A. ventricosus* lipids on phagocytosis of RAW264.7 cells. Values are shown as the mean ± SD (*n* = 3). * *p* < 0.05, indicating statistical differences compared to cells treated with RPMI. M1 = macrophage phenotypes related to immunological stimulating activity; AVL = *A. ventricosus* lipids.

Macrophage phagocytosis after treatment with *A. ventricosus* lipids was analyzed using FITC-labeled dextran. As illustrated in Figure 3, LPS dramatically boosted phagocytic capacity as compared to RPMI, the negative control. A reagent control experiment with DMSO showed a slight stimulation of phagocytosis and no difference compared to RPMI. Moreover, *A. ventricosus* lipids (100–300 µg/mL) reinforced the phagocyte activities observed in RAW264.7 cells, depending on the dose.

### 2.4. Effects of A. ventricosus Lipids on NO Production and iNOS Expression

**Figure 4:**
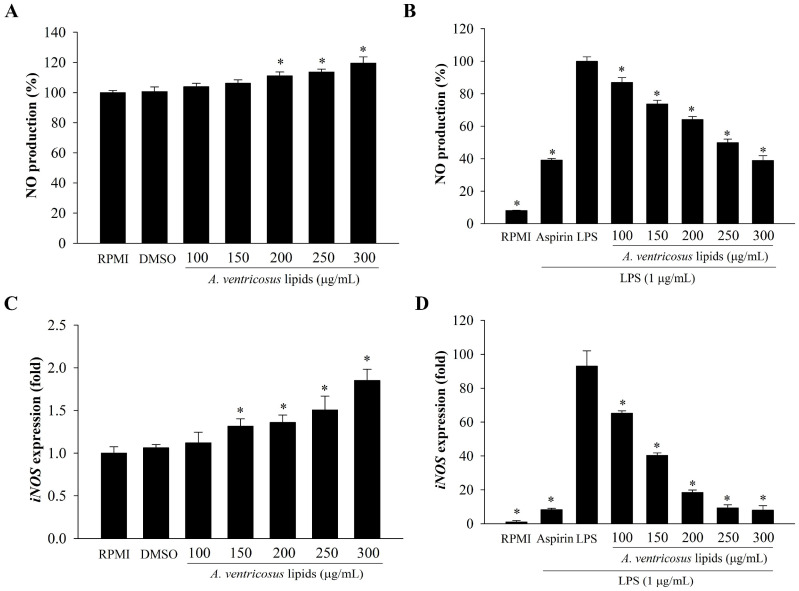
Effects of *A. ventricosus* lipids on NO production and *iNOS* expression. The production of NO was measured in RAW264.7 cells (**A**) and in LPS-stimulated RAW264.7 cells (**B**). The mRNA levels of *iNOS* in RAW264.7 cells (**C**) and in LPS-stimulated RAW264.7 cells (**D**) are shown. Values are shown as the mean ± SD (*n* = 3). * *p* < 0.05, indicating statistical differences compared to cells treated with the control (RPMI or LPS).

The effects of *A. ventricosus* lipids on macrophage immunity were tested by measuring NO production in macrophages treated with these lipids at varying concentrations (100–300 µg/mL). As shown in Figure 4A, *A. ventricosus* lipids promoted NO secretion of macrophages in a dose-varying manner. When compared with RPMI, treatments with DMSO and *A. ventricosus* lipids at 100 or 150 µg/mL slightly and non-significantly increased NO secretion of macrophages, whereas *A. ventricosus* lipids at 200, 250, and 300 µg/mL significantly increased NO secretion. Furthermore, the anti-inflammatory effect of *A. ventricosus* lipids (100–300 µg/mL) dramatically decreased LPS-induced NO generation by 86.95–38.89%, depending on the dose. The highest dose of *A. ventricosus* lipids, at 300 µg/mL, had a significant suppressive effect on LPS-induced NO generation (38.89 ± 2.94%), similar to the result of aspirin (39.08 ± 0.98%), a positive drug (Figure 4B). As shown in Figure 4C, *A. ventricosus* lipids substantially boosted *iNOS* expression, whereas these lipids suppressed LPS-induced *iNOS* expression (Figure 4D).

### 2.5. Effects of A. ventricosus Lipids on Cytokine Expression

**Figure 5:**
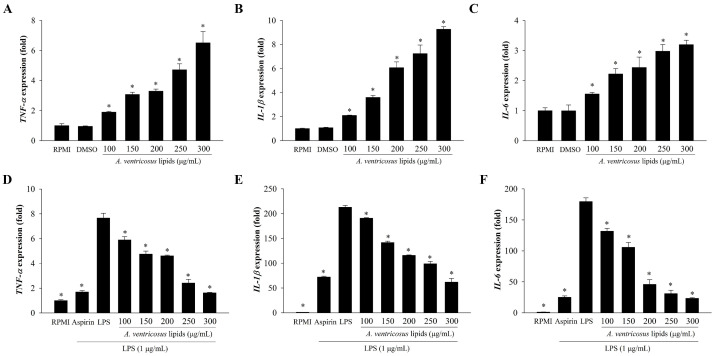
Effects of *A. ventricosus* lipids on cytokine expression. The mRNA levels of *TNF-α*, *IL-1β*, and *IL-6* in RAW264.7 cells (**A**–**C**) and in LPS-stimulated RAW264.7 cells (**D**–**F**) are shown. Values are shown as the mean ± SD (*n* = 3). * *p* < 0.05, indicating statistical differences compared to cells treated with the control (RPMI or LPS).

The immunoregulatory effects of *A. ventricosus* lipids on the expression of immune-related genes are shown in Figure 5. The expression levels of *IL-1β*, *IL-6*, and *TNF-α* were substantially enhanced by *A. ventricosus* lipids in a dose-varying manner when compared with those in RPMI, as shown in Figure 5A–C. The mRNA expression of three cytokines (*IL-1β*, *IL-6*, and *TNF-α*) in LPS-treated cells were also significantly increased when compared with those in RPMI. However, *A. ventricosus* lipids effectively suppressed the LPS-induced expression of *IL-1β*, *IL-6*, and *TNF-α* in a dose-varying manner (Figure 5D–F).

### 2.6. Effects of A. ventricosus Lipids on NF-κB and MAPK Activation

**Figure 6:**
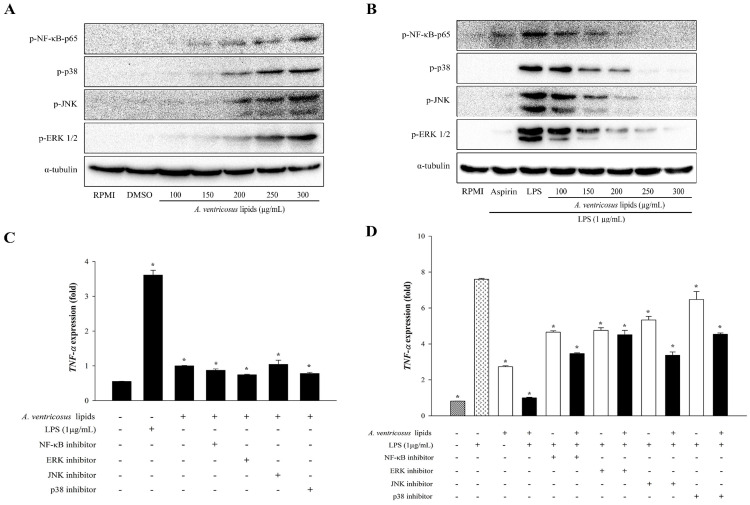
Effect of *A. ventricosus* lipids on NF-κB and MAPK activation. Phosphorylation levels of proteins of the NF-κB and MAPK signaling pathways in RAW264.7 cells (**A**) and in LPS-induced RAW264.7 cells (**B**). Effects of specific inhibitors of NF-κB and MAPK on *A. ventricosus* lipid-induced *TNF-α* expression in RAW264.7 cells (**C**) and in LPS-stimulated RAW264.7 cells (**D**). Values are shown as the mean ± SD (*n* = 3). * *p* < 0.05, indicating statistically significant differences compared to cells treated with the control (RPMI or LPS).

To determine whether *A. ventricosus* lipids modulate immune signaling pathways, their effects on the expression levels of NF-κB and MAPK proteins were explored using a Western blotting assay. As a control for the immune-enhancement function, RPMI was used. *A. ventricosus* lipids substantially increased the phosphorylation of NF-κB-p65 in RAW264.7 cells in a dose-varying manner. The phosphorylation levels of ERK1/2, JNK, and p38 were also increased by *A. ventricosus* lipids (Figure 6A). Conversely, to identify the regulation of anti-inflammatory function of *A. ventricosus* lipids, LPS-stimulated cells were used. *A. ventricosus* lipids (100–300 µg/mL) obviously suppressed the phosphorylation of NF-κB p65. Depending on the dose, *A. ventricosus* lipids also strongly reduced JNK, ERK, and p38 activation induced by LPS (Figure 6B).

### 2.7. Effects of A. ventricosus Lipids on TNF-α Expression after Co-Treatment with Specific Inhibitors via NF-κB and MAPK Activation

To confirm that the MAPK and NF-κB pathways were involved in *A. ventricosus* lipid-induced macrophage activation, specific NF-κB, JNK, ERK, and p38 MAPK inhibitors were used along with *A. ventricosus* lipids (300 µg/mL). To identify which immune-enhancement signaling pathway was involved, the levels of *TNF-α* expression were measured by real-time qPCR (Figure 6C). *A. ventricosus* lipids or LPS alone increased the expression levels of NF-κB, p38, JNK, and ERK1/2 when compared with the control (RPMI). *TNF-α* expression was significantly decreased after treatment with *A. ventricosus* lipids in the presence of NF-κB and MAPK compared to that after treatment with LPS, whereas *A. ventricosus* lipids increased *TNF-α* expression in the presence of NF-κB and MAPK activation compared to RPMI (Figure 6C). Conversely, to confirm which immune-signaling pathway was involved in the anti-inflammation effect, LPS was used to stimulate the cells (Figure 6D). Compared to LPS alone, co-treatment of *A. ventricosus* lipids with NF-κB, JNK, ERK, and p38 MAPK inhibitors markedly decreased *TNF-α* expression: 3.46 ± 0.04-fold by a specific inhibitor for NF-κB, 4.51 ± 0.24-fold by a specific inhibitor for p38 MAPK, 3.37 ± 0.19-fold by a specific inhibitor for JNK, and 4.54 ± 0.07-fold by a specific inhibitor for ERK.

### 2.8. Effects of A. ventricosus Lipids on LPS-Induced Cell Surface Molecule Expression

**Figure 7:**
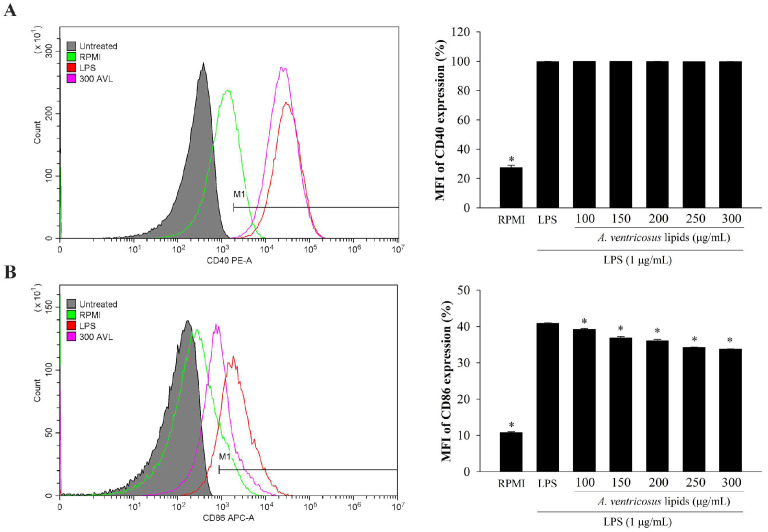
Effect of *A. ventricosus* lipids on expression of CD40 (**A**) and CD86 (**B**) in LPS-stimulated RAW264.7 cells. Values are shown as the mean ± SD (*n* = 3). * *p* < 0.05, indicating statistically significant differences when compared with cells treated with LPS. M1 = macrophage phenotypes related to immunological stimulating activity. AVL = *A. ventricosus* lipids.

Figure 7 show cell surface molecule associated with inflammation regulation in LPS-stimulated cells. Results revealed that expression level of cell surface molecule CD40 on macrophages was significantly upregulated after treatment with LPS, whereas CD40 expression did not differ significantly from that of the group treated with LPS followed by *A. ventricosus* lipids (Figure 7A). However, *A. ventricosus* lipids at 100–300 µg/mL significantly decreased LPS-induced expression of CD86 by 39.25–33.80% (Figure 7B). These findings suggested that *A. ventricosus* lipids triggered cell surface activation via the CD86 molecule.

## 3. Discussion

In the present study, lipids were extracted from *A. ventricosus* eggs. Their FA profile was then evaluated using a GC assay after sample saponification and derivatization to their corresponding methyl esters. Among them, palmitic acid, oleic acid, DHA, and EPA stood out. EPA and DHA are major fatty acids found in eggs of several fish species including tuna, white seabream, Atlantic bonito, cuttlefish, lumpfish, and Atlantic salmon [13,14,30]. The immunomodulatory effect of DHA on RAW264.7 cells was associated with the release of NO and the protein expression of cytokines (iNOS, IL-1β, IL-6, IL-10, IL-12, TNF-α, IFN-γ, and TGF-β) by stimulating GPR120, C-Raf, and MAPKs of the NF-κB p65 pathway [31]. Many previous studies have shown its immunomodulating effect in fish eggs [20,21,32,33]. However, the mechanisms of the immune-regulating actions of *A. ventricosus* lipids remain unknown. In this study, the anti-inflammatory and immunostimulatory effects of *A. ventricosus* lipids on RAW264.7 macrophages were elucidated and their possible mechanisms of action were examined.

Macrophages are immune cells that generate cytotoxic and inflammatory substances such as NO and release cytokines in reaction to foreign infections [34]. NO is a biomarker that regulates immune function and inflammatory mediators. It may contribute to the release of various hormones implicated in immunological regulation [35,36]. Our results showed that *A. ventricosus* lipids had the capacity to increase NO generation in RAW264.7 cells for immune enhancement. Consistent with previous reports showing that syntheses of pro-inflammatory mediators such as NO and PGE_2_ are induced by iNOS and COX-2 enzymes, respectively [7], *A. ventricosus* lipids also induced *iNOS* expression. Additionally, previous studies have indicated that fish lipids can lead to the expression of inflammatory cytokines (such as *TNF-α*, *IL-1β*, and *IL-6*) and mediators (*iNOS*) in RAW264.7 cells [20,21,37], consistent with similar the immune-regulating effects of another compound previously reported [38,39]. Our results also revealed that *A. ventricosus* lipids increased the expression of *IL-1β*, *IL-6*, and *TNF-α* in RAW264.7 cells. Conversely, the secretion of NO and the expression levels of *iNOS* gene were reduced in LPS-stimulated cells due to the anti-inflammatory effects of *A. ventricosus* lipids. *A. ventricosus* lipids reduced the expression of immune-associated genes. They also decreased *iNOS* expression in LPS-stimulated cells, similar to previous reports [9,11,12]. These results suggest that *A. ventricosus* lipids may regulate immunity, such as through immune enhancement and anti-inflammation, by releasing pro-inflammatory mediators and cytokines through macrophages depending on the conditions.

Activation of NF-κB and MAPK cellular signaling plays important roles in macrophage immunity [5,6,11]. Once the macrophage is stimulated by diverse stimulants such as bacteria and LPS, the p65 subunit essential for the NF-κB family becomes activated and transfers from the cytoplasm to the nucleus [40]. In addition, multiple biological functions such as cell development, differentiation, proliferation, apoptosis, the reaction to oxidative stress, and inflammatory responses are known to be regulated by MAPK, such as ERK, JNK, and p38 [41]. Lipids extracted from fish eggs have been reported to be involved in inflammation by activating the NF-κB and MAPK pathways [20,21,37]. Our study also found that *A. ventricosus* lipids substantially regulated the immune system by increasing or decreasing the phosphorylation of NF-κB- and MAPK-associated proteins, depending on the conditions of immunity enhancement and anti-inflammation. In addition, the analysis of *TNF-α* expression after treatment with specific NF-κB, JNK, ERK, and p38 MAPK inhibitors showed that *A. ventricosus* lipids were associated with its regulation via the NF-κB and MAPK activation, similar to previous reports [4,7,11]. Taken together, these findings suggest that *A. ventricosus* lipids can reduce inflammation by inhibiting the NF-κB and MAPK pathways.

Phagocytosis is one of macrophages’ representative immunological responses. It is activated at the initial phases of immunity and inflammatory [42]. Recently, it has been reported that *Paecilomyces lilacinus* exopolysaccharide can significantly increase the phagocytic capability of RAW264.7 cells using FITC-labeled dextran [43]. In our current study, to measure phagocytosis ability, flow cytometry was used. The results showed that *A. ventricosus* lipids triggered phagocytosis, indicating that *A. ventricosus* lipids might activate macrophages, increasing their ability to phagocytose. Moreover, prior research has revealed that CD40 and CD86 lead to the immune function of the macrophage surface induced by natural compounds [8,44]. It has been shown that T-cell responses are able to modulated with either pro-inflammatory or regulatory effector functions by differential expression of co-stimulatory molecules on antigen-presenting cells [45]. Our results showed that CD86, a cell surface molecule, was decreased in LPS-stimulated macrophages due to the anti-inflammatory effect of *A. ventricosus* lipids. These results indicate that *A. ventricosus* lipids may influence the phagocytic activity and activation of LPS-induced macrophages by decreasing CD86 expression.

Overall, these results indicate that *A. ventricosus* lipids exhibit immunomodulatory effects by inhibiting or enhancing the immune system via macrophages, with their inhibition or enhancement depending on the presence of LPS, which thus shapes the immune condition of macrophage cells. Numerous natural substances possess immunomodulatory agents that exhibit a broad spectrum of immune-enhancing and anti-inflammatory activities, which are utilized to either increase or decrease an organism’s susceptibility to invading antigens [46,47,48].

## 4. Materials and Methods

### 4.1. Samples

*A. ventricosus* was obtained from Jumunjin Fish Market in Gangneung, Gangwon-do, Korea. Eggs collected from *A. ventricosus* were freeze-dried, blended, and kept at −20 °C.

### 4.2. Preparation of A. ventricosus Lipids

The Bligh and Dyer method [49] was carried out to extract lipids from prepared *A. ventricosus* eggs. In brief, solutions were produced by mixing 4.5 g of dried materials with 30 mL of chloroform: methanol (1:2, *v*/*v*), then adding chloroform and distilled water. After centrifuging the mixture at 3000 rpm for 10 min, the aqueous solution was collected and filtered through a filter paper (Whatman No.2, Whatman, Maidstone, UK) and a 0.2 µm PTFE membrane (CHM lab group, Barcelona, Spain). After that, the filtered solution was concentrated using an IKA RV 10 Digital V-C rotary evaporator (IKA, Staufen, Germany) and a 12 position N-EVAP nitrogen evaporator (Organomation, Berlin, MA, USA). The lipid yield obtained from *A. ventricosus* eggs was 15.48 ± 0.68%. These lipids were then extracted using a dimethyl sulfoxide (DMSO) solution for use in cell culture experiments.

### 4.3. Determination of Fatty Acid Compositions

*A. ventricosus* lipids were subjected to extraction of fatty acid methyl esters (FAMEs) through a one-step procedure involving hydrolysis, extraction, and methylation [50,51]. FAMEs were quantified using an Agilent 7890 gas chromatograph equipped with a flame-ionization detector (Agilent Technologies, Santa Clara, CA, USA) and an Agilent J&W GC Column (30 m × 0.32 mm I.D., 0.25 µm film thickness; Agilent Technologies, Santa Clara, CA, USA). Helium was the primary gas used. Temperatures for the injector and detector were continually maintained at 250 °C, while the oven was initially set to 150 °C. It was subsequently raised to 230 °C at a rate of 3.5 °C/min and held at this temperature for 10 min. FA peaks were identified by comparing their retention times to those of various FAME standards.

### 4.4. Cell Culture and Treatments

Cells used in this study were RAW264.7 macrophages (Korea Cell Line Research Foundation, Seoul, Republic of Korea). Cells were cultured in media containing RPMI-1640, 10% fetal bovine serum (FBS), and 1% penicillin/streptomycin (P/S) in an incubator at 37 °C with 5% CO_2_. Treatment groups were as follows: DMSO (1%), aspirin (200 µg/mL), and *A. ventricoseus* lipids (at varying concentrations of 100, 150, 200, 250, and 300 µg/mL). All samples were diluted and used by adding 1% FBS and 1% PS to RPMI-1640 medium without phenol red. Cultured cells received 100 µL of each reagent. After 1 h, the anti-inflammatory model was treated with 100 µL of LPS (1 µg/mL), while 100 µL of medium was added to the immune enhancement model and then incubated for another 24 h.

### 4.5. Assay of Cell Viability

Cells (1 × 10^6^ cells/mL) were placed in a 96-well plate and then treated with different samples. After 24 h of incubation, the cell culture medium was removed and the water-soluble tetrazolium salt (WST) solution of the EZ-Cytox Cell Viability Assay Kit (Daeil Lab Service, Seoul, Republic of Korea) was added. The cells were then incubated for 1 h at 37 °C. The absorbance was measured at 450 nm using an EPOCH 2 microplate reader (Agilent BioTek, Santa Clara, CA, USA).

### 4.6. Measurement of NO Production

To assess the immune-regulating efficacy of lipids, the NO level was determined using Greiss reagent (Promega, Madison, WI, USA). Supernatants of treated cells (100 µL) were combined with Griess solution (100 µL), which contained Greiss reagents A and B (1% sulfanilamide in 5% phosphoric acid and 0.1% *N*-1-napthylethylenediamine dihydrochloride in water). After incubating at room temperature for 10 min, the absorbance at 540 nm was recorded.

### 4.7. RNA Isolation and Real-Time qPCR

Following cell culture and sample treatment, the total RNA was prepared using TRI reagent^®^ (Molecular Research Center, Inc., Cincinnati, Ohio, USA). After cell lysis, the solution was transferred to a fresh microtube and homogenized using a vortex with 200 µL of chloroform. The supernatant was centrifuged at 13,000 rpm for 10 min at 4 °C before being transferred to a fresh microtube and incubated with isopropanol at 4 °C for 1 h. The pellet was obtained after incubation and centrifuged at 13,000 rpm for 10 min at 4 °C before being washed three times with 70% ethanol. The pellet of total RNA was dissolved in DEPC-treated water. The RNA purity and concentration were then assessed. A High-Capacity cDNA Reverse Transcription Kit (Applied Biosystems, Waltham, MA, USA) was used to perform reverse transcription of the RNA to cDNA. Amplification reactions contained cDNA (5 ng), TB Green Premix Ex Taq II (Takara Bio Inc., Kusatsu, Japan), ROX Reference Dye, and 7.5 µg of forward and reverse primers to assess the expression of immune genes. The primer sequences were as follows: IL-1β (5′-GGGCCTCAAAGGAAAGAATC-3′ and 5′-TACCAGTTGGGGAACTCTGC-3′); IL-6 (5′-AGTTGCCTTCTTGGGAC TGA-3′ and 5′-CAGAATTGCCATTGCACAAC-3′); TNF-α (5′-ATGAGCACAGAAAGCA TGATC-3′ and 5′-TACAGGCTTGTCACTCGAATT-3′); iNOS (5′-TTCCAGAATCCCTGGACAAG-3′ and 5′-TGGTCAAACTCTTGGGGTTC-3′); β-actin (5′-CCACAGCTGAGAG GGAAATC-3′ and 5′-AAGGAAGGCTGGAAAAGAGC-3′). A QuantStudio™ 3 FlexReal-Time PCR System (Applied Biosystems, Waltham, MA, USA) was used to conduct the experiment and analyze the obtained results.

### 4.8. Western Blotting Assay

Cells (2 × 10^6^ cells/mL) were collected and incubated on ice for 30 min in a cell lysis buffer supplemented with protease and phosphatase inhibitors. The cell lysate was centrifuged at 13,000 rpm for 10 min at 4 °C, and the supernatant was collected. The protein concentration was determined using a Pierce™ BCA protein assay (Thermo Fisher Scientific, Waltham, MA, USA). Proteins from each treatment were separated by SDS-polyacrylamide gel electrophoresis before they were subsequently transferred to polyvinylidene fluoride membranes (Merck, Kenilworth, NJ, USA). The membranes were blocked with 5% skim milk in TBST buffer for 1 h at room temperature. Following primary antibody incubation against phospho-p38 MAPK, phospho-SAPK/JNK, phospho-p44/42 MAPK (ERK1/2), phospho-NF-κB p65 (Cell signaling Technology, Danvers, MA, USA), and α-tubulin (Abcam, Cambridge, UK), the membranes were then incubated with secondary antibodies such as goat anti-rabbit IgG(H+L)-HRP (GenDEPOT, Katy, TX, USA). Detection of protein signals was performed with Pierce^®^ ECL Plus Western Blotting Substrate (Thermo Fisher Scientific, Waltham, MA, USA) and a Bio-Rad ChemiDoc XRS+ system (Bio-Rad, Hercules, CA, USA).

### 4.9. Inhibition of NF-κB and MAPK Using Specific Inhibitors

To further investigate signaling pathways through which *A. ventricosus* lipids activated macrophages, RAW264.7 cells were pretreated with either 100 nM of NF-κB activation inhibitor (Calbiochem, Burlington, MA, USA) for 3 h or 20 µM of MAPK inhibitors including ERK, JNK inhibitor II, and p38 MAP kinase inhibitor (Calbiochem, Burlington, MA, USA) for 1 h. These inhibitor-treated cells were washed with 1×PBS buffer twice. Cells were then treated with *A. ventricosus* lipids at 300 µg/mL and LPS. After 24 h of incubation, the cells were then used to isolate RNA. *TNF-α* expression was determined using real-time qPCR, as described above. 

### 4.10. Phagocytic Uptake of Macrophages

Cells treated with *A. ventricosus* lipids were harvested and incubated with 1 mg/mL of FITC-dextran (Sigma-Aldrich, St. Louis, MO, USA) at 37 °C for 1 h. After three washes with cold 1×PBS buffer, the cells were resuspended with cold FACS buffer (2% FBS and 0.1% sodium azide in 1×PBS buffer). Flow cytometry was then performed using a Cyto FLEX Flow Cytometer (Beckman Coulter, Inc., Brea, CA, USA).

### 4.11. Analysis of Expression of Cell Surface Molecules

Sample-treated cells were collected and washed with cold FACS buffer. The cells were blocked with purified rat IgG (Thermo Fisher Scientific, Waltham, MA, USA) for 10 min. Then, the cells were incubated with specific antibodies of CD40-PE and CD86-APC (Invitrogen, USA) in combination with their isotype control antibodies for 20 min. Flow cytometry analysis was conducted using a CytoFLEX Flow Cytometer and the CytEx-pert program (Beckman Coulter, Inc., Brea, CA, USA).

### 4.12. Statistical Analysis

All analysis of data were carried out using IBM SPSS statistics 23 software. One-way ANOVA was applied, immediately following Duncan’s multiple-range test at *p* < 0.05. Results are presented as the mean ± SD.

## 5. Conclusions

In this study, lipids extracted from *A. ventricosus* eggs contained a high concentration of PUFAs (54.73%) such as DHA and EPA. *A. ventricosus* lipids exhibited immune-regulating effects on RAW264.7 macrophages by modulating the expression of inflammatory mediators (NO and iNOS) and cytokines (TNF-α, IL-1β, and IL-6) through the NF-κB and MAPK pathways. Furthermore, *A. ventricosus* lipids significantly improved the phagocytic function and suppressed cell surface molecule CD86 expression. These results suggest that lipids extracted from *A. ventricosus* eggs might have potential as immunomodulatory functional substances.

## Data Availability

The datasets used and/or analyzed during the current study are available from the corresponding author upon reasonable request.

## References

[1] Choi Y.H., Jin G.Y., Li G.Z., Yan G.H. (2011). Cornuside suppresses lipopolysaccharide-induced inflammatory mediators by inhibiting nuclear factor-kappa B activation in RAW 264.7 macrophages. Biol. Pharm. Bull..

[2] Fujiwara N., Kobayashi K. (2005). Macrophages in Inflammation. Curr. Drug Targets Inflamm. Allergy.

[3] Florean C., Dicato M., Diederich M. (2022). Immune-modulating and anti-inflammatory marine compounds against cancer. Semin. Cancer Biol..

[4] Xie Y., Wang L., Sun H., Wang Y., Yang Z., Zhang G., Jiang S., Yang W. (2019). Polysaccharide from alfalfa activates RAW264.7 macrophages through MAPK and NF-kB signaling pathways. Int. J. Biol. Macromol..

[5] Fang Q., Wang J.-F., Zha X.-Q., Cui S.-H., Cao L., Luo J.-P. (2015). Immunomodulatory activity on macrophage of a purified polysaccharide extracted from *Laminaria japonica*. Carbohydr. Polym..

[6] Ko M.N., Hyun S.B., Ahn K.J., Hyun C.-G. (2022). Immunomodulatory effects of *Abelmoschus esculentus* water extract through MAPK and NF-κB signaling in RAW264.7 cells. Biotechnol. Notes.

[7] Lee M.-S., Kwon M.-S., Choi J.-W., Shin T., No H.K., Choi J.-S., Byun D.-S., Kim J.-I., Kim H.-R. (2012). Anti-inflammatory activities of an ethanol extract of *Ecklonia stolonifera* in lipopolysaccharide-stimulated RAW264.7 murine macrophage cells. J. Agricul. Food Chem..

[8] Lim J., Rod-in W., Monmai C., Jang A.Y., Choi J., Park W.-J. (2022). In vitro immune-enhancement and anti-inflammatory effects of fatty acids extracted from the *Halocynthia aurantium* gonad on RAW264.7 macrophages. Nutrients.

[9] Li M., Zhang L., Cai R.-L., Gao Y., Qi Y. (2012). Lipid-soluble extracts from *Salvia miltiorrhiza* inhibit production of LPS-induced inflammatory mediators via NF-κB modulation in RAW264.7 cells and perform anti-inflammatory effects in vivo. Phytother. Res..

[10] Son H.J., Eo H.J., Park G.H., Jeong J.B. (2021). *Heracleum moellendorffii* root extracts exert immunostimulatory activity through TLR2/4-dependent MAPK activation in mouse macrophages, RAW264.7 cells. Food Sci. Nutr..

[11] Wang T., Wu F., Jin Z., Zhai Z., Wang Y., Tu B., Yan W., Tang T. (2014). Plumbagin inhibits LPS-induced inflammation through the inactivation of the nuclear factor-kappa B and mitogen activated protein kinase signaling pathways in RAW 264.7 cells. Food Chem. Toxicol..

[12] Kim K.N., Heo S.J., Yoon W.J., Kang S.M., Ahn G., Yi T.H., Jeon Y.J. (2010). Fucoxanthin inhibits the inflammatory response by suppressing the activation of NF-kB and MAPKs in lipopolysaccharide-induced RAW 264.7 macrophages. Eur. J. Pharmacol..

[13] Cejas J.R., Almansa E., Villamandos J.E., Badía P., Bolaños A., Lorenzo A. (2003). Lipid and fatty acid composition of ovaries from wild fish and ovaries and eggs from captive fish of white sea bream (*Diplodus sargus*). Aquaculture.

[14] Garaffo M.A., Vassallo-Agius R., Nengas Y., Lembo E., Rando R., Maisano R., Dugo G., Giuffrida D. (2011). Fatty acids profile, atherogenic (IA) and thrombogenic (IT) health lipid indices, of raw roe of Blue Fin Tuna (*Thunnus thynnus* L.) and their salted product “Bottarga”. Food Nutr. Sci..

[15] Huynh M.D., Kitts D.D., Hu C., Trites A.W. (2007). Comparison of fatty acid profiles of spawning and non-spawning Pacific herring, *Clupea harengus pallasi*. Comp. Biochem. Physiol. B Biochem. Mol. Biol..

[16] Wall R., Ross R.P., Fitzgerald G.F., Stanton C. (2010). Fatty acids from fish: The anti-inflammatory potential of long-chain omega-3 fatty acids. Nutr. Rev..

[17] Mullen A., Loscher C.E., Roche H.M. (2010). Anti-inflammatory effects of EPA and DHA are dependent upon time and dose-response elements associated with LPS stimulation in THP-1-derived macrophages. J. Nutr. Biochem..

[18] Holub D.J., Holub B.J. (2004). Omega-3 fatty acids from fish oils and cardiovascular disease. Mol. Cell. Biochem..

[19] Torrejon C., Jung U.J., Deckelbaum R.J. (2007). n-3 Fatty acids and cardiovascular disease: Actions and molecular mechanisms. Prostaglandins Leukot Essent Fat. Acids.

[20] Lim J.H., Choi G.S., Monmai C., Rod-in W., Jang A.Y., Park W.J. (2021). Immunomodulatory activities of *Ammodytes personatus* egg lipid in RAW264.7 cells. Molecules.

[21] Choi G.S., Lim J.H., Rod-In W., Jung S.K., Park W.J. (2022). Anti-inflammatory properties of neutral lipids, glycolipids, and phospholipids isolated from *Ammodytes personatus* eggs in LPS-stimulated RAW264.7 cells. Fish Shellfish Immunol..

[22] Orlov A.M., Tokranov A.M. (2008). Specific features of distribution, some features of biology, and the dynamics of catches of smooth lumpsucker *Aptocyclus ventricosus* (Cyclopteridae) in waters of the Pacific Ocean off the Kuril Islands and Kamchatka. J. Ichthyol..

[23] Solomatov S.F., Orlov A.M. (2018). Smooth lumpsucker *Aptocyclus ventricosus* in the northwestern Sea of Japan: Distribution and some life history traits. Fish. Aquat. Life.

[24] Okazaki T., Stevenson D.E., Kai Y., Ueda Y., Hamatsu T., Yamashita Y. (2020). Genetic population structure and demographic history of a pelagic lumpsucker, *Aptocyclus ventricosus*. Environ. Biol. Fish.

[25] Kim I.-S., Park H.-J., Jeong B.-Y., Moon S.-K. (2020). Food components characteristics of the muscles and roes of Smooth Lumpsucker *Aptocyclus ventricosus* and Korai bikunin *Liparis ingens* from the East sea, Korea. Korean J. Fish. Aquat. Sci..

[26] Yates C.M., Calder P.C., Ed Rainger G. (2014). Pharmacology and therapeutics of omega-3 polyunsaturated fatty acids in chronic inflammatory disease. Pharmacol. Ther..

[27] Swanson D., Block R., Mousa S.A. (2012). Omega-3 fatty acids EPA and DHA: Health Bbenefits throughout life. Advances in Nutrition.

[28] Jeon M., Kim H., Park J.J., Kim J.W., Lee J.S. (2016). Ultrastructure of Integument of the Smooth Lumpsucker, *Aptocyclus ventricosus* (Pallas, 1769)(Teleostei: Cyclopteridae). Korean J. Ichthyol..

[29] Kobayashi K. (1962). Larvae of the smooth lumpsucker, *Aptocyclus ventricosus* (Pallas), with discussion on revision of the taxonomy of the species. Bull. Fac. Fish. Hokkaido Univ..

[30] Rincón-Cervera M.Á., Suárez-Medina M.D., Guil-Guerrero J.L. (2009). Fatty acid composition of selected roes from some marine species. Eur. J. Lipid Sci. Technol..

[31] Han L., Yu J., Chen Y., Cheng D., Wang X., Wang C. (2018). Immunomodulatory activity of docosahexenoic acid on RAW264.7 cells activation through GPR120-mediated signaling pathway. J. Agric. Food Chem..

[32] Chalamaiah M., Hemalatha R., Jyothirmayi T., Diwan P.V., Uday Kumar P., Nimgulkar C., Dinesh Kumar B. (2014). Immunomodulatory effects of protein hydrolysates from rohu (*Labeo rohita*) egg (roe) in BALB/c mice. Food Res. Int..

[33] Tseng C.-C., Chu T.-W., Danata R.H., Risjani Y., Shih H.-T., Hu S.-Y. (2020). Hepcidin-expressing fish eggs as a novel food supplement to modulate immunity against pathogenic infection in Zebrafish (*Danio rerio*). Sustainability.

[34] Taylor P.R., Martinez-Pomares L., Stacey M., Lin H.H., Brown G.D., Gordon S. (2004). Macrophage receptors and immune recognition. Annu. Rev. Immunol..

[35] Boscá L., Zeini M., Través P.G., Hortelano S. (2005). Nitric oxide and cell viability in inflammatory cells: A role for NO in macrophage function and fate. Toxicology.

[36] Coleman J.W. (2001). Nitric oxide in immunity and inflammation. Int. Immunopharmacol..

[37] Rod-in W., Monmai C., Shin I.-S., You S., Park W.J. (2020). Neutral lipids, glycolipids, and phospholipids, isolated from Sandfish (*Arctoscopus japonicus*) eggs, exhibit anti-inflammatory activity in LPS-stimulated RAW264.7 cells through NF-κB and MAPKs pathways. Mar. Drugs.

[38] Eo H.J., Park Y., Kwon H.Y., Park G.H. (2022). Immune-enhancing effects of *Hibiscus syriacus* roots in RAW264.7 macrcophages. Food Agric. Immunol..

[39] Shen C.-Y., Yang L., Jiang J.-G., Zheng C.-Y., Zhu W. (2017). Immune enhancement effects and extraction optimization of polysaccharides from *Citrus aurantium* L. var. amara Engl. Food Funct..

[40] Tak P.P., Firestein G.S. (2001). NF-kB: A key role in inflammatory diseases. J. Clin. Investig..

[41] Thalhamer T., McGrath M.A., Harnett M.M. (2008). MAPKs and their relevance to arthritis and inflammation. Rheumatology.

[42] Wang G., Zhu L., Yu B., Chen K., Liu B., Liu J., Qin G., Liu C., Liu H., Chen K. (2016). Exopolysaccharide from *Trichoderma pseudokoningii* induces macrophage activation. Carbohydr. Polym..

[43] He C., Lin H.Y., Wang C.C., Zhang M., Lin Y.Y., Huang F.Y., Lin Y.Z., Tan G.H. (2019). Exopolysaccharide from *Paecilomyces lilacinus* modulates macrophage activities through the TLR4/NF-κB/MAPK pathway. Mol. Med. Rep..

[44] Eckhardt A., Harorli T., Limtanyakul J., Hiller K.-A., Bosl C., Bolay C., Reichl F.-X., Schmalz G., Schweikl H. (2009). Inhibition of cytokine and surface antigen expression in LPS-stimulated murine macrophages by triethylene glycol dimethacrylate. Biomaterials.

[45] Tripathi S., Bruch D., Kittur D.S. (2008). Ginger extract inhibits LPS induced macrophage activation and function. BMC Complement. Altern. Med..

[46] Kumar A., Sawhney G., Kumar Nagar R., Chauhan N., Gupta N., Kaul A., Ahmed Z., Sangwan P.L., Satheesh Kumar P., Yadav G. (2021). Evaluation of the immunomodulatory and anti-inflammatory activity of Bakuchiol using RAW 264.7 macrophage cell lines and in animal models stimulated by lipopolysaccharide (LPS). Int. Immunopharmacol..

[47] Yun Y., Han S., Park E., Yim D., Lee S., Lee C.-K., Cho K., Kim K. (2003). Immunomodulatory activity of betulinic acid by pro-ducing pro-inflammatory cytokines and activation of macrophages. Arch. Pharm. Res..

[48] Apostolova E., Lukova P., Baldzhieva A., Katsarov P., Nikolova M., Iliev I., Peychev L., Trica B., Oancea F., Delattre C. (2020). Immunomodulatory and anti-inflammatory effects of Fucoidan: A review. Polym..

[49] Bligh E.G., Dyer W.J. (1959). A rapid method of total lipid extraction and purification. Can. J. Biochem. Physiol..

[50] Park W.J., Kothapalli K.S.D., Lawrence P., Tyburczy C., Brenna J.T. (2009). An alternate pathway to long-chain polyunsaturates: The *FADS2* gene product Delta8-desaturates 20:2n-6 and 20:3n-3. J. Lipid Res..

[51] Garces R., Mancha M. (1993). One-step lipid extraction and fatty acid methyl esters preparation from fresh plant tissues. Anal. Biochem..

